# 个体化治疗时代多靶点药物究竟何去何从——多靶点药物治疗非小细胞肺癌最新临床进展综述

**DOI:** 10.3779/j.issn.1009-3419.2011.11.08

**Published:** 2011-11-20

**Authors:** 彩存 周

**Affiliations:** 200433 上海，同济大学医学院附属上海市肺科医院肿瘤科 Department of Oncology, Shanghai Pulmonary Hospital, Affiliated to Tongji University School of Medicine, Shanghai 200433, China

**Keywords:** 肺肿瘤, 多靶点药物, 舒尼替尼, 索拉非尼, 凡德他尼, Crizotinib, Lung neoplasms, Multi-targeted tyrosine kinase inhibitor, Sunitinib, Sorafenib, Vandetanib, Crizotinib

## Abstract

目前在非小细胞肺癌治疗中，多靶点药物占有举足轻重的地位。近年来随着多项Ⅲ期临床结果的陆续公布，多靶点药物的疗效可谓喜忧参半。在个体化治疗的大方向下，如何发挥多靶点药物的最佳疗效，的确值得我们探索。本文综述了近年来多靶点药物治疗非小细胞肺癌的最新临床进展，希望能有所启发。

## Abstract (en)

Multi-targeted tyrosine kinase inhibitors (TKIs) play important roles in the treatment of non-small cell lung cancer (NSCLC). Recently, results of several large phase Ⅲ studies have been published, showing bittersweet efficacy of multi-targeted TKIs. In the era of personalized therapy, optimal use of multi-targeted TKIs is worthy of further investigation. In this review, we summarized current clinical development and future direction of multi-targeted TKIs in NSCLC..

近年来，靶向药物的应用改变了非小细胞肺癌（non-small cell lung cancer, NSCLC）传统治疗的模式。从1962年Stanley Cohen教授首次发现表皮生长因子（epidermal growth factor, EGF），到2008年IPASS研究奠定了表皮生长因子受体酪氨酸激酶抑制剂（epidermal growth factor receptor tyrosine kinase inhibitors, EGFR-TKIs）在*EGFR*突变型晚期NSCLC治疗中的重要地位，直至2010年公布的OPTIMAL研究更是首次将晚期NSCLC患者的中位无进展生存期（progression-free survival, PFS）延长至1年以上^[1, 2]^。

但是，多靶点药物在前几年的喧嚣过后，其在NSCLC领域的临床试验结果往往不尽如人意。似乎对于NSCLC患者来说，这类具有良好理论基础的药物走入了一个进退两难的境地。本文就NSCLC领域多靶点药物的临床新进展进行综述，以期为该类药物的未来发展方向提供参考。

## 1 多靶点药物的抗肿瘤作用机制

多靶点药物在NSCLC领域之所以被寄予厚望与其独特的理论基础及抗肿瘤作用机制是密不可分的。首先，对于单靶点药物而言，虽然其对于特定NSCLC患者的疗效卓越，但对于多数患者来说疗效较差。出现这种情况的理论基础在于NSCLC是具有高度异质性的恶性肿瘤，其活化的肿瘤信号通路纷繁复杂，且纵横交错。因此，单靶点药物在封闭肿瘤关键驱动信号通路的同时也激活了肿瘤的逃逸机制，最终的结果是肿瘤细胞可能通过其它旁路再次激活其增殖^[3]^。由此可见，在确保安全、低毒的前提下，尽可能多地抑制肿瘤信号通路不失为一种明智之举。其次，表 1列出了常见NSCLC多靶点药物的作用靶点，从中可以看出，相对于仅针对肿瘤细胞的单靶点药物而言，大部分多靶点药物也可通过抑制血管内皮生长因子受体（vascular endothelial growth factor receptor, VEGFR）信号通路对肿瘤生长的微环境进行抑制，即同时具有抗血管生成和抗细胞增殖的双重作用机制。

**1:** 多靶点药物作用靶点列表 IC_50_ value of different signal pathway by multi-targeted TKIs

Kinase inhibition	Sunitinib (IC_50_, nmol/L)	Motesanib (IC_50_, nmol/L)	Sorafenib (IC_50_, nmol/L)	Vandetanib (IC_50_, nmol/L)
VEGFR-1	2	2	--	1, 600
VEGFR-2	4	3	90	40
VEGFR-3	17	6	20	110
PDGFR-*α*	69	84	--	
PDGFR-*β*	39	--	57	1, 100
Raf	--	91	6	--
c-Kit	1	8	68	> 20, 000
FLT3	8	--	58	
EGFR	> 10, 000	> 3, 000	> 10, 000	500
RET	224	59	47	130

临床前的研究数据已经证实，多靶点药物可通过抑制VEGF依赖的血管内皮活性从而发挥其抗肿瘤血管生成样作用。更进一步来说，通过对VEGFR信号通路的抑制，以舒尼替尼为代表的多靶点药物不仅可使肿瘤微血管退化，而且可抑制新生肿瘤血管的生成，并且减少肿瘤血管的渗出，最终阻断肿瘤细胞的血供，导致肿瘤细胞液化坏死^[4-11]^。

另一方面，多靶点药物直接针对肿瘤细胞信号通路的靶点在NSCLC中同样可发挥一定的抗肿瘤细胞增殖的作用。例如，凡德他尼可抑制EGFR信号通路，舒尼替尼可抑制血小板衍生生长因子受体α（platelet-derived growth factor receptor α, PDGFRα）信号通路，索拉非尼可抑制RAS/RAF信号通路。

从作用机制上看，作为具有双重作用机制的多靶点药物，似乎理论上的疗效可优于单靶点药物，下面将阐述一下现实的临床数据。

## 2 多靶点药物最新临床进展

### 2.1 舒尼替尼（Sunitinib, Sutent, SU11248）

从化学结构上看，舒尼替尼是一类新型吲哚酮类口服多靶点酪氨酸激酶抑制剂，其抗肿瘤活性已在包括肾细胞癌、胃肠道间质瘤、软组织肉瘤、甲状腺癌中得到证实。对舒尼替尼在NSCLC中抗肿瘤活性的探索，源于其对包括H226 NSCLC在内的多种实体肿瘤细胞株良好的抑瘤作用^[12]^。Socinski等^[13]^在化疗失败的NSCLC患者中进行的Ⅱ期临床研究证实了舒尼替尼单药的抗肿瘤活性。该研究共入组了63例传统化疗失败的晚期NSCLC患者，给予舒尼替尼50 mg/d，用药4周停药2周（4/2方案）。与基线相比，共有15例患者的肿瘤缩小 > 30%。按RECIST标准评价，获得部分缓解（partial response, PR）的患者为7例，另有18例患者疾病稳定（stable disease, SD）超过8周; 疾病获得控制患者的中位治疗持续时间为22.1周。在所有患者中，中位PFS为12周，中位总生存期（overall survival, OS）为23.4周。大部分舒尼替尼相关的不良反应为轻度-中度，且多可耐受，最常见的1度-2度/3度-4度不良反应为乏力（41%/27%）、疼痛/肌肉痛（43%/17%）和恶心/呕吐（40%/10%）; 血液系统的不良反应主要为3度-4度中性粒细胞减少（5%）和血小板减少（5%）。对于舒尼替尼而言，另一重要问题为给药剂量，因此与该试验同时进行的另一项Ⅱ期临床试验则在相同的入组人群中给予37.5 mg/d持续治疗的方案，其结果详见表 2。

**2:** 舒尼替尼治疗NSCLC 50 mg/d用药4周停药2周方案*vs* 37.5 mg/d持续给药方案 50 mg/d 4 weeks on, 2 weeks off *vs* 37.5 mg continuous daily dose regimen of sunitinib in NSCLC

		Sunitinib 50 mg/d 4/2 (*n*=63)	37.5 mg/d CDD (*n*=47)
Efficacy	ORR (%)	11.1	2.1
	PFS (week)	12.0	12.3
	OS (week)	23.4	37.1
Adverse events (%, grade 1-2/3-4)	Fatigue	41/29	43/17
	Pain	43/17	47/2
	Nause/Vomiting	43/10	38/2
	Stomatitis	40/3	29/2
	Hypertension	6/5	19/9
	Hemoptysis	NR	15/2
	Neutropenia	44/5	NR/9
	Thrombocytopenia	49/5	NR/0
CDD: continous daily dose; PFS: progression-free survival; OS: overall survival; ORR: objective response rate; NR: not reported.			

基于Ⅱ期临床试验的结果，舒尼替尼开始了其Ⅲ期临床研究。而2010年ESMO已正式公布了其随机双盲Ⅲ期临床试验（SUN 1087）的最终结果。该研究主要针对一线化疗失败的二线NSCLC患者，旨在对比舒尼替尼联合厄洛替尼与厄洛替尼单药的疗效，力图通过靶向药物的联合应用改变NSCLC的二线治疗模式。对总体人群而言，患者在一线或二线铂类化疗失败后，相对于单药厄洛替尼而言，接受舒尼替尼与厄洛替尼的联合治疗可明显改善其PFS（3.6个月*vs* 2.0个月，*P*=0.002, 3），并提高客观有效率（*P*=0.047, 1）^[14]^。最终可能由于后续治疗的原因，总体生存无明显差异（OS：9.0个月*vs* 8.5个月）。就安全性而言，联合治疗组多数的不良反应均在可预测、可控、可管理的范围之内。

值得大家关注的是，今年世界肺癌大会公布了在SUN 1087亚裔患者的亚组分析结果^[15]^。在103例亚裔患者中，舒尼替尼联合厄洛替尼明显改善了晚期NSCLC患者的总生存，中位OS分别为未达到（95%CI：13.4个月-未达到）*vs* 9.4个月（95%CI：7.5个月-15.4个月），HR达到了0.532（*P*=0.010, 1），死亡风险下降了46.8%;两组患者的中位PFS分别为31.2周*vs* 15.2周（*P*=0.088, 9）; 而客观缓解率（objective response rate, ORR）几乎达到了单药治疗组的3倍（38.5% *vs* 13.7%）。虽然由于研究开始较早且为二/三线治疗的原因，该研究缺乏*EGFR*基因突变状态的数据，但是类似于这类通过大型临床试验中的亚组分析找到优势人群的探索思路似乎已逐渐成为靶向药物未来的发展方向。而舒尼替尼在亚裔人群中的疗效究竟如何值得我们进一步探索。

### 2.2 凡德他尼（Vandetanib, Zactima, ZD6474）

凡德他尼与舒尼替尼略有不同，其化学结构中含有苯胺喹唑啉结构，该类化合物是目前活性最高、选择性最好的酪氨酸激酶抑制剂。凡德他尼的主要靶点包括EGFR、VEGFR、RET。理论上同时抑制EGFR和VEGFR这两条在NSCLC中最为重要的信号通路似乎能获得较好的临床疗效。而较早的一项Ⅱ期临床试验结果似乎也印证了这一理论的存在。Natale等^[16]^进行的凡德他尼与吉非替尼二线治疗NSCLC头对头比较的临床试验显示，凡德他尼和吉非替尼的中位PFS分别为11.0周*vs* 8.1周（HR=0.69, *P*=0.025）。由于该Ⅱ期研究允许疾病进展后相互交叉至对方组，OS差异并未达到统计学意义（7.4个月*vs* 6.1个月）。

得到上述Ⅱ期临床试验的鼓舞，三项大型Ⅲ期临床试验同时对凡德他尼在NSCLC中的疗效进行了研究。但是，随后公布的结果却令人大跌眼镜。首先公布的ZODIAC研究^[17]^共入组了1, 400例晚期NSCLC患者，研究二线比较了凡德他尼联合多西他赛和多西他赛单药的疗效。凡德他尼虽然延长了患者的PFS（中位PFS：4.0个月*vs* 3.2个月，HR=0.79，*P* < 0.001），但是OS未获得具有统计学意义的改善（HR=0.91）。之后，凡德他尼联合培美曲塞（ZEAL）^[18]^和凡德他尼联合厄洛替尼（ZEST）^[19]^研究结果的公布更是让人失望，ZEAL或ZEST分别与培美曲塞或厄洛替尼单药组相比，虽然ORR有所提高，但PFS、OS均未获得具有统计学意义的改善。同样遗憾的是上述Ⅲ期临床研究均未就*EGFR*基因状态进行分层分析，故未能进一步发现凡德他尼的可能获益人群。凡德他尼对EGFR的IC_50_值为500 nmol/L^[20]^，对EGFR信号通路的抑制率过低，可能也是凡德他尼在上述临床试验中未获成功的原因。

### 2.3 索拉非尼（Sorafenib, Nexevar, BAY439006）

索拉非尼具有高度活性的芳基脲结构，而该结构对RAS/RAF激酶具有高度的抑制效应^[21]^。因此，研发之初索拉非尼被认为是丝氨酸/苏氨酸激酶抑制剂，随着研究的深入，发现其对VEGFR、PDGFRβ、c-Kit信号通路中的酪氨酸激酶也具有抑制作用。

基于贝伐珠单抗联合化疗在NSCLC一线治疗中的成功，索拉非尼分别联合泰素/卡铂（ESCAPE）和吉西他滨/顺铂（NEXUS）进行了两项Ⅲ期临床试验^[22, 23]^。但遗憾的是，与凡德他尼类似，其结果并不尽如人意。且索拉非尼联合治疗组中鳞癌患者的死亡率明显高于单纯化疗组。2010年ESMO公布了NEXUS的最终结果，将132例鳞癌患者排除后，索拉非尼联合吉西他滨/顺铂用于NSCLC一线治疗仍未改善患者的ORR和OS（主要终点），分别为28% *vs* 26%（*P*=0.27）和379 d *vs* 376 d。因此，正在进行的索拉非尼单药三/四线治疗NSCLC的MISSION研究^[24]^已经结束入组，该研究试图在三/四线NSCLC治疗中找到多靶点药物的地位，它的最终结果值得我们期待。

### 2.4 其它多靶点药物

阿西替尼（AG013736）是一种多靶点药物，其主要的作用靶点为VEGFR、PDGFRβ和c-KIT，是目前对VEGFR信号通路抑制率最高的酪氨酸激酶抑制剂^[25]^，已在多种实体肿瘤中显示出良好的抗肿瘤活性。基于今年ASCO公布的一项Ⅲ期临床试验结果，阿西替尼已成为肾癌二线治疗的新标准^[26]^。在一项阿西替尼单药治疗晚期NSCLC的Ⅱ期临床研究^[27]^中，共有3例患者获得疾病缓解（3/32, 9%），中位PFS和OS分别为4.9个月和14.8个月，显示了其在NSCLC患者中良好的抗肿瘤活性和安全性。目前，正在同时进行的三项Ⅱ期研究正在进一步探索阿西替尼联合化疗（AGILE 1030：阿西替尼联合泰素/卡铂; AGILE 1039：阿西替尼联合培美曲塞/顺铂; AGILE 1038：阿西替尼联合吉西他滨/顺铂）治疗非鳞NSCLC的疗效和安全性。

Motesanib抑制的靶点主要包括VEGFR、PDGFR、KIT和RET。一项早期的Ib期临床试验结果^[28]^显示，Motesanib联合泰素/卡铂和/或Panitumumab治疗晚期NSCLC安全性良好，54例患者中3例发生剂量限制性毒性，分别为1例4度肺栓塞（50 mg qd剂量组）、2例3度深静脉栓塞（125 mg qd剂量组）。其余常见不良反应为乏力、腹泻、高血压和厌食。在正在进行的一项Ⅲ期临床中，在鳞癌患者中Motesanib联合泰素/卡铂治疗的咯血发生率增高，故该Ⅲ期临床研究目前只能纳入非鳞癌患者，最终结果有待进一步的研究。

## 3 非小细胞肺癌：多靶点药物何去何从？

如上所述，虽然多靶点药物在NSCLC领域的疗效已取得一定的进展，但是如果坚持在非选择性NSCLC患者中继续进行探索的话，其结果可想而知。正如表 3所示，在非选择性人群中，多靶点药物与单靶点药物单药治疗NSCLC无论是ORR、PFS还是OS均无明显差异。因此，如何找到多靶点药物的适合人群显得尤为重要。

**3:** 多靶点药物*vs*单靶点药物单药治疗NSCLC疗效对比 Outcome of multi-targeted *vs* mono-targeted TKIs in NSCLC

Study	Drug	*n*	Median PFS (week)	Median OS (week)	ORR (%)	DCR (%)
Socinski	Sunitinib	63	11.3	24	9.5%	52%
Blumenschein	Sorafenib	52	11.9	29	0%	59%
Ronald B	Vandetanib	83	11	24	8%	45%
BR21	Erlotinib	488	9.4	28.7	6.9%	43.0%
Interest	Gefitinib	659	9.4	32.6	9.7%	45.8%
DCR: disease control rate.						

首先，从肿瘤驱动基因的角度上考虑，尽管肺癌存在一些驱动基因，但真正对临床有指导作用的只有*EGFR*突变与*EML4-ALK*融合基因^[29]^，多数患者癌变驱动基因目前仍不明确。特别是对于EGFR-TKIs耐药的患者，此时肿瘤关键驱动信号通路已经发生改变，依赖交横错落的信号通路，致癌信号通过多通道重新发挥了致癌作用。因此，在耐药机制不明确的情况下，多靶点药物在EGFR-TKIs失败后的解救治疗中的确具有一定优势。在保证安全性的前提下，尽可能抑制肿瘤信号通路似乎是提高疗效的捷径。

而今年ASCO^[30]^和世界肺癌大会^[31]^报道的肺癌肿瘤驱动基因筛选结果也从另一侧面解释了这一选择的现实性。无论是腺癌还是鳞癌，即使通过高通量的筛选技术，仍分别存在46%和37%的患者无法确定其肿瘤驱动基因。对于这部分患者，多靶点药物应该可以发挥更大的作用。

其次，与单靶点药物不同，多靶点药物通常可抑制的信号通路众多，在发挥关键抑制作用信号通路未知的情况下，试图寻找其靶点无论从实际操作层面还是从理论层面均存在一定的困难。抗血管生成治疗在NSCLC中的靶点至今仍未有定论，而多靶点药物通常均同时具有抗血管生成和抗细胞增殖的双重作用。在部分有效的NSCLC患者中，往往是双重抑制模式并存，其所导致的结果常可能产生一个包含多种（而非单一）因子的复合预测模型。这也是多靶点药物优势人群探索不得不面对的问题。另外，就多靶点药物抗肿瘤作用的理论基础而言，针对多个靶向信号通路，使用单一药物无法保证对所有靶点均达到最佳抑制浓度。这就意味着如果血药浓度存在差异的话，其靶点的抑制效果可能也存在着一定的差异。这一效应的产生也干扰着多靶点药物在NSCLC患者疗效中的均一性。

关于这一点，去年美国癌症研究学会（American Association for Cancer Research, AACR）年会公布的SUN 1058试验^[32]^可能会给大家带来一定的启示。该研究对舒尼替尼联合厄洛替尼对比单药厄洛替尼二/三线治疗NSCLC的一项Ⅱ期临床试验进行了生物标志物分析。其探索基本囊括了与舒尼替尼相关所有靶点的表达情况以及*EGFR*、*KRAS*的突变状态分析。结果显示，舒尼替尼的疗效与*EGFR*、*KRAS*的突变状态无明显相关性。在组织学标本指标中，PDGFRα mRNA低表达患者的疗效优于PDGFRα mRNA高表达的患者（HR=0.386, *P* < 0.05）。该研究对血清蛋白分子标志物也进行了探索，并首先发现类似sVEGFR-3、VEGFC以及sVEGFR-2的变化与舒尼替尼疗效的相关性。通过SUN 1087试验，可以猜测或许只有通过大范围的靶点筛查，多靶点药物才能为NSCLC患者带来惊喜。

## 4 总结

在NSCLC患者中，多靶点药物的疗效并未得到一致的认可。基于肿瘤驱动基因的概念，针对驱动机制未明的EGFR-TKIs耐药的患者，或许能提高多靶点药物在NSCLC中的疗效^[33-35]^。从另一方面来说，基于分子标志物的个体化治疗已成为将来NSCLC的发展方向。如何为合适的患者选择合适的药物，达到疗效/不良反应的最优化组合，已成为肿瘤领域医师探索的热点。多靶点药物只有找到属于自己的优势人群，才是其未来发展的真正出路所在。
